# Delayed diagnosis of skeletal dysplasia in a girl with cartilage-hair hypoplasia

**DOI:** 10.1210/jcemcr/luag102

**Published:** 2026-04-28

**Authors:** Petra Loid, Eleni P Kotanidou, Vasiliki Rengina Tsinopoulou, Assimina Galli-Tsinopoulou, Outi Mäkitie

**Affiliations:** Children's Hospital, University of Helsinki and Helsinki University Hospital, Helsinki 00290, Finland; Research Program for Clinical and Molecular Metabolism, University of Helsinki, Helsinki 00014, Finland; Folkhälsan Research Center, Genetics Research Program, Helsinki 00290, Finland; Department of Molecular Medicine and Surgery, Karolinska Institutet, Stockholm 17176, Sweden; 2nd Department of Pediatrics, School of Medicine, Faculty of Health Sciences, Aristotle University of Thessaloniki, AHEPA University General Hospital, Thessaloniki 54636, Greece; 2nd Department of Pediatrics, School of Medicine, Faculty of Health Sciences, Aristotle University of Thessaloniki, AHEPA University General Hospital, Thessaloniki 54636, Greece; 2nd Department of Pediatrics, School of Medicine, Faculty of Health Sciences, Aristotle University of Thessaloniki, AHEPA University General Hospital, Thessaloniki 54636, Greece; Children's Hospital, University of Helsinki and Helsinki University Hospital, Helsinki 00290, Finland; Research Program for Clinical and Molecular Metabolism, University of Helsinki, Helsinki 00014, Finland; Folkhälsan Research Center, Genetics Research Program, Helsinki 00290, Finland; Department of Molecular Medicine and Surgery, Karolinska Institutet, Stockholm 17176, Sweden; Clinical Genetics, Karolinska University Hospital, Stockholm 17164, Sweden

**Keywords:** cartilage hair hypoplasia, short stature, chondrodysplasia, diagnosis

## Abstract

Short stature is the most common reason for endocrinological investigations during childhood. With the expanding spectrum of recognized genetic causes for abnormal growth, establishing the correct diagnosis is often challenging. We present a 12-year-old girl who was first evaluated for short stature (<3rd percentile) and short fingers at age 2 years. Skeletal radiographs showed mild long bone metaphyseal abnormalities. She had slightly thin hair, unilateral elbow dislocations, and genu varum but no hematologic abnormalities or signs of immunodeficiency. Growth hormone testing and other endocrine investigations were normal. At age 12 years, when her height was −3.0 SD score, skeletal dysplasia was considered, and genetic testing revealed compound heterozygosity for 2 *RMRP* variants (n.97_98dup and n.-25_-12dup), confirming the diagnosis of cartilage-hair hypoplasia (CHH), a recessive skeletal dysplasia. Retrospective review of early radiographs showed that diagnostic features were present already at the first assessment. The described patient is the first reported case of CHH in Greece. The delayed diagnosis led to several unnecessary investigations. This case highlights the importance of considering skeletal dysplasia in children with short stature. Early diagnosis of CHH is important for careful follow-up because of increased risk for immunodeficiency and malignancies.

## Introduction

Short stature is a common reason for referrals to pediatric endocrinologists and often prompts investigations to identify potential underlying endocrinological or genetic condition. Recent advances have expanded the spectrum of known genetic causes for growth abnormalities [1]. Despite this, the identification of rare genetic causes, such as skeletal dysplasia, remains challenging.

Skeletal dysplasias comprise 771 conditions associated with 552 genes [1]. Cartilage-hair hypoplasia (CHH, OMIM #250250) is a rare autosomal recessive metaphyseal chondrodysplasia characterized by severe short-limbed short stature, metaphyseal dysplasia, hypoplastic hair, immunodeficiency, hematological abnormalities, and predisposition to cancer and gastrointestinal dysfunction [2]. CHH is most prevalent in the Finnish and Amish populations but has been reported in multiple other populations [3-5].

CHH was the first human disorder linked to variants in a long noncoding RNA gene [6]. The disease is caused by biallelic variants in the untranslated *RMRP* gene, the RNA component of the RNase MRP ribonucleoprotein complex [6]. This complex participates in ribosomal RNA processing, mRNA cleavage, telomere maintenance, immune regulation, and cell-cycle control [2, 7, 8].

The diagnosis of CHH is based on clinical and radiographic findings. Short-limbed short stature is usually evident at birth or in early childhood. The growth failure is progressive because of slow infantile growth and poor pubertal growth spurt, resulting in severe short stature in adulthood (median adult height 123 cm for females, 131 cm for males) [9]. However, growth retardation may be mild and some patients can have normal childhood height [10]. Radiographs show variable degree of metaphyseal dysplasia, short tubular bones, short phalanges with cone-shaped epiphyses, bowed femora and tibiae, and lumbar lordosis [2]. Diagnosing CHH can be difficult because its clinical and radiographic features vary in severity and hair hypoplasia may be absent. This may result in delayed diagnosis.

We report the first case of CHH in Greece. This girl with short stature underwent extensive laboratory and radiographic examinations from age 2 years, and the specific diagnosis was delayed until age 12 years. The correct diagnosis was made after expert consultation, and single-gene testing confirmed the suspected diagnosis. This case highlights the need for clinical awareness of skeletal dysplasia, such as CHH, in children and adolescents with short stature.

## Case presentation

**Figure 1:**
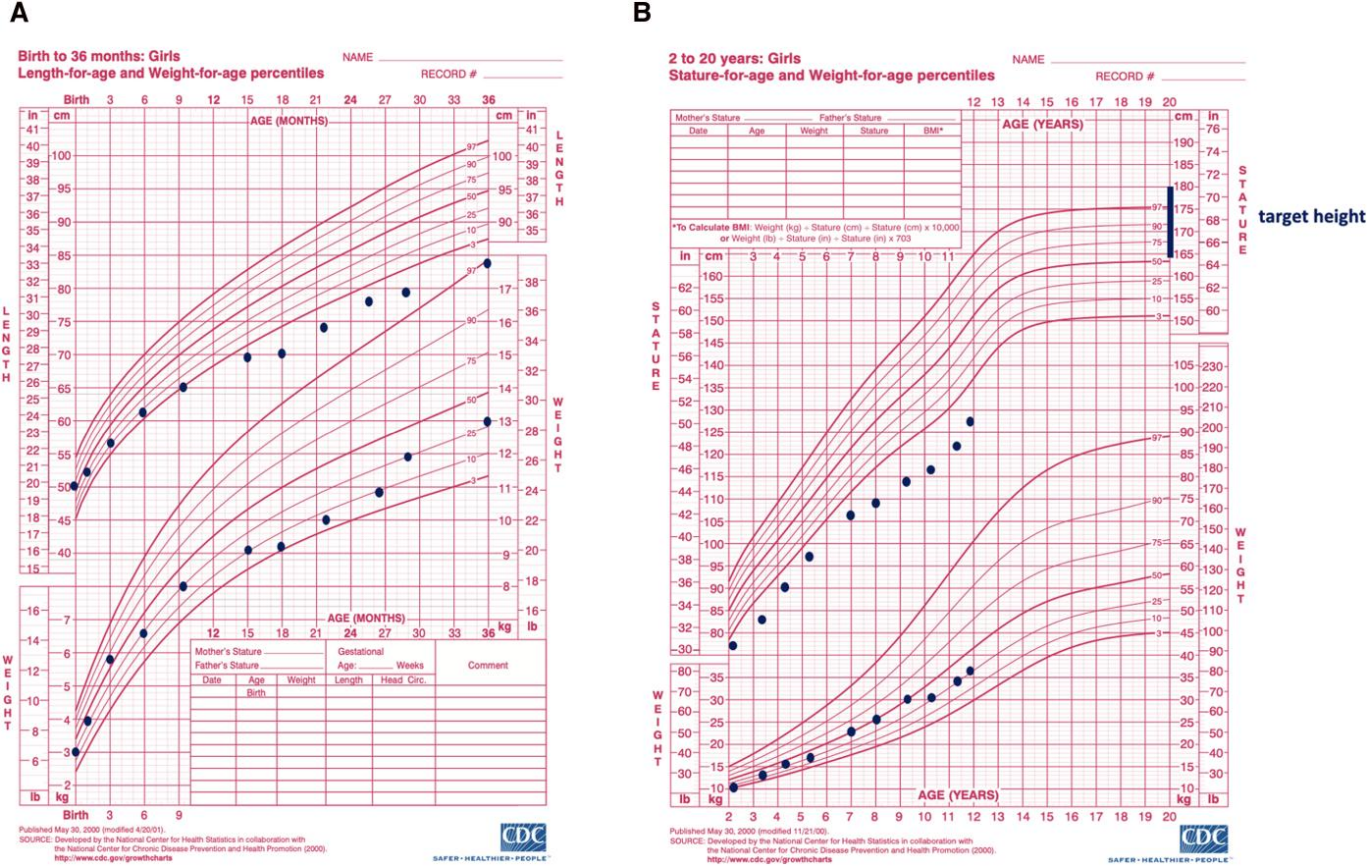
The growth curves of the proband on Centers for Disease Control and Prevention reference length/height and weight for age percentile charts: (A) birth to 36 months (B) from 2 to 20 years. The curves indicate normal birth measurements but early deceleration of longitudinal growth during the first year, followed by relatively stable growth pattern until prepuberty when the growth further deviates from normal.

**Figure 2:**
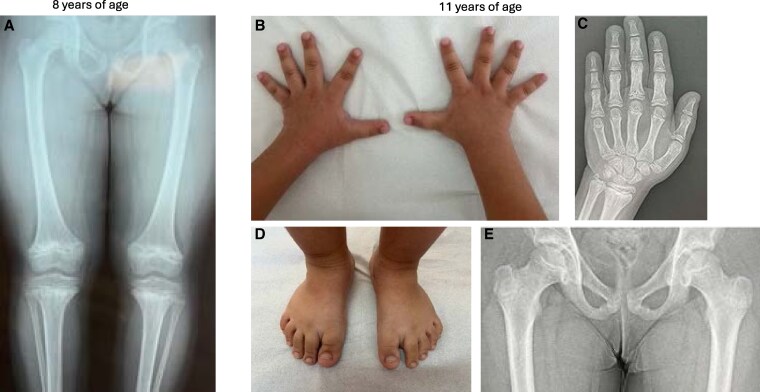
Lower limb radiograph at 8 years shows metaphyseal widening and irregularity at the knees, both in distal femurs and proximal tibiae, whereas the proximal femurs show normal femoral necks and metaphyses; epiphyses are normal in shape (A). At 11 years, hands show short fingers (B). Hand radiograph shows short metacarpals and phalanges with metaphyseal flaring and irregularity, cone-shaped phalangeal epiphyses (4th and 5th finger proximal phalanx) (C). Bone age development is uneven, with absent radial sesamoid bone but premature closure of some distal phalangeal growth plates (C). Feet are short and broad with short digits (D). Hip radiograph shows very mild metaphyseal irregularity and normal secondary ossification center (E).

The proband is a 12-year-old Greek female born to healthy nonconsanguineous parents at 38 weeks of gestation by cesarean section. Birth length was 50 cm (50th-75th percentile) and weight 3150 g (25th-50th percentile). The mother's height was 172 cm and father's height 187 cm. She had 3 healthy siblings. She was first evaluated for short stature at 2.3 years when her height was 77.5 cm (<3rd percentile), weight 11.05 kg (10th-25th percentile), and head circumference 48.5 cm (25th-50th percentile). Her growth curves on Centers for Disease Control and Prevention reference charts are presented in Fig. 1. She had short arms and brachydactyly. Thyroid function, GH provocation tests, serological celiac disease testing, and karyotype were normal. Magnetic resonance imaging of the pituitary gland was normal. Skeletal survey at 2.3 years of age showed mild abnormality of the long bone metaphyses, mainly at the proximal metaphyses of arms, femur, and knees. She had multiple unilateral elbow dislocations and bilateral genu varum. Her hair was fine and silky. At ages 8 and 11 years, skeletal radiographs revealed radiological findings that argued for molecular expert advice: wide and irregular metaphyses of the long bones especially at the knees and wrists, whereas hips were less affected. Similar metaphyseal changes were present in the metacarpals and phalanges. In addition, cone-shaped phalangeal epiphyses were observed together with phalangeal shortening and premature closure of the distal phalangeal growth plates (Fig. 2). Spinal and skull radiographs were normal.

At 11.9 years of age, her height was 127.8 cm (−3.0 SD score), weight 37 kg (25th-50th percentile), body mass index 22.7 kg/m2 (>95th percentile), arm span 138 cm, and sitting height 57.7%. Her Tanner stage was pubic hair stage 3, breast development stage 2 with presence of axillary hair, but no menarche. Bone age was 13 years. Dual-energy X-ray absorptiometry showed normal bone mineral density. Hematological and biochemical evaluations, including IGF-1 and gonadotropin levels were normal (Table 1). She had no history of recurrent or serious infections.

**Table 1:** Laboratory investigations at CHH diagnosis

Parameters	Values	Reference range
Fasting glucose	82 mg/dL (4.5 mmol/L)	<99 mg/dL (<5.5 mmol/L)
Insulin	11 μIU/mL (76.3 pmol/L)	2.6-17 μIU/mL (18 -118 pmol/L)
HbA1c	5.2%	<5.7%
IGF-1	386.5 ng/mL (50.52 nmol/L)	134-602 ng/mL (17.5-78.7 nmol/L)
FSH	3.0 mIU/mL	1.5-12.8 mIU/mL
LH	1.1 mIU/mL	0.1-12 mIU/mL
Estradiol	10 pg/mL (36.7 pmol/L)	7-60 pg/mL (25 pmol/L-220.3 pml/L)
Calcium	10 mg/dL (2.49 mmol/L)	9.2-10.5 mg/dL (2.3-2.6 mmol/L)
Phosphate	4.6 mg/dL (1.5 mmol/L)	4.1-5.9 mg/dL (1.3-1.9 mmol/L)
Magnesium	2.2 mg/dL (0.9 mmol/L)	2.1-2.8 mg/dL (0.9-1.2 mmol/L)
Alkaline phosphate	183 U/L	141-460 U/L
25-OH-Vitamin D	20 ng/mL (50 nmol/L)	20-80 ng/mL (50-200 nmol/L)
PTH	28.9 pg/mL 3.1 pmol/L	10-65 pg/mL (1-6.8 pmol/L)

For parameters with conventional values, International System of Units (SI) values are provided in parentheses.

Abbreviation: HbA1c, hemoglobin A1c.

## Diagnostic assessment

**Figure 3:**
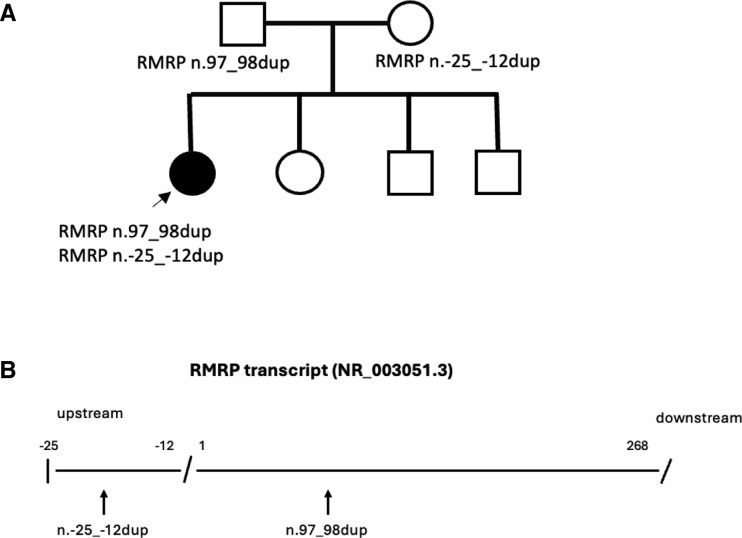
(A) Pedigree of the family with the genetic test results. (B) Illustration of the location of the RMRP variants n.97_98dup and n.-25_-12dup identified in our study.

After review of clinical and radiographic findings with a skeletal dysplasia expert (O.M.), a targeted *RMRP* single-gene test was performed at Blueprints Genetics (Espoo, Finland), at age 12 years. Two previously reported heterozygous pathogenic and likely pathogenic *RMRP* variants were identified (NR_003051.3): n.97_98dup and n.-25_-12dup. Both parents were heterozygous carriers of 1 of the variants (Fig. 3A), confirming recessive inheritance. The location of the 2 *RMRP* variants is illustrated in Fig. 3B.

The patient's findings were regarded consistent with the diagnosis of CHH. Retrospective radiographic review showed the presence of the diagnostic metaphyseal changes already at age 2 years.

## Treatment

No specific medical treatment was initiated. Management focused on multidisciplinary supportive care and clinical follow-up.

## Outcome and follow-up

Parents received genetic counselling, and the patient was referred to thorough immunological evaluation and regular pediatric hematology and oncology follow-up. The total Ig and IgG subclasses were normal (Table 2). Complete blood count did not reveal abnormal findings; the total white blood cell count and the proportions and absolute numbers of lymphocytes, granulocytes, eosinophils, and basophils were normal. Peripheral blood immunophenotype study with flow cytometry separation to examine leukocyte populations was also normal. An adequate immune response for previous vaccinations was documented for measles, rubella, and mumps. In contrast, varicella zoster virus IgG was negative, despite 2 doses of vaccination, and antibody to hepatitis B surface antigen was negative.

**Table 2:** Immunological investigations after CHH diagnosis

Parameters	Values	Reference range
Total serum IgG	1010 mg/dL (67.3 μmol/L)	639-1349 mg/dL (42.6-89.9 μmol/L)
Total serum IgA	171 mg/dL (10.6 μmol/L)	70-312 mg/dL (4.37- 19.5 μmol/L)
Total serum IgM	114 mg/dL (1.17 μmol/L)	56-352 mg/dL (0.57- 3.62 μmol/L)
Total serum IgE	81.9 IU/mL (81.9 IU/mL)	2.06-195.2 IU/mL (2.06-195.2 IU/mL)
Serum IgG subclass 1	544 mg/dL (5.44 g/L)	280-1030 mg/dL (2.80-10.30 g/L)
Serum IgG subclass 2	237.2 mg/dL (2.37 g/L)	66-502 mg/dL (0.66-5.02 g/L)
Serum IgG subclass 3	109 mg/dL (1.09 g/L)	11.5-105.3 mg/dL (0.115-1.053 g/L)
Serum IgG subclass 4	37.2 mg/dL (0.372 g/L)	1.0-121.9 mg/dL (0.01-1.219 g/L)
C3	115 mg/dL (1.15 g/L)	85-160 mg/dL (0.85-1.6 g/L)
Antinuclear autoantibodies	<1:40	<1:160
Autoantibodies targeting double-stranded DNA	<10	<100

For parameters with conventional values, International System of Units (SI) values are provided in parenthesis.

Abbreviation: C3, Complement Component 3.

## Discussion

We describe a Greek patient with short-limbed short stature, brachydactyly, mild metaphyseal dysplasia, and mild hair hypoplasia, whose diagnosis of CHH was delayed until age 12 years.

CHH is a rare disorder, and therefore many clinicians may not be familiar with the clinical features, especially in populations where the disease is uncommon. This can result in delayed referral for appropriate genetic testing. CHH should be considered in patients with short stature or findings of metaphyseal dysplasia. Other suggestive findings include brachydactyly, joint hypermobility, hypoplastic hair, recurrent or severe infections, anemia, and intestinal dysfunction [2]. High phenotypic variability complicates diagnosis. Our patient had normal birth length and less severe short stature compared to typical CHH patients [2, 9]. However, her growth pattern—postnatal growth retardation with no catch-up growth—is typical for skeletal dysplasia. Hair hypoplasia and hematological and immunological abnormalities, apart from poor vaccination response, were absent, demonstrating variable and often subtle nature of extraskeletal manifestations in CHH.

Radiological evaluation is crucial in differential diagnosis of skeletal dysplasias. A comprehensive skeletal survey is necessary to determine which bones are affected. Typical radiographic findings in CHH include metaphyseal flaring and irregularities, especially at the knees [2]. However, the skeletal changes may be absent during infancy or may be present only for a limited period in childhood. Furthermore, interpreting radiographic findings can be challenging. In our patient, mild metaphyseal changes were observed on skeletal radiographs already at 2 years of age, particularly at the knees. Additionally, brachydactyly and cone-shaped epiphyses, features typical of CHH, were also present. These features can be detected on normal bone age radiograph. Skeletal dysplasias are rare disorders and it is advisable to seek guidance from experts, as was done in this case. The European Reference Network (ERN), also has a specialized network, ERN on rare bone disorders, which helps to identify centers and experts in rare bone diseases.

Genetic testing for *RMRP* should be considered for the diagnosis of CHH. Genetic diagnosis is important and gene panel testing is the most common method for the genetic diagnosis of skeletal dysplasias when the clinical diagnosis is unclear. In settings with limited resources and without access to specialized networks, phenotypic assessment and skeletal surveys are essential. Optional diagnostic strategies include targeted Sanger sequencing of *RMRP* or whole-exome or whole-genome sequencing. Genetic testing of the *RMRP* gene should be designed to include both the transcribed and promoter region of *RMRP* and, if needed, duplication/deletion analysis of *RMRP*. Because *RMRP* gene is an untranslated long noncoding RNA, it does not have exons and pathogenic variants are easily missed in normal exome sequencing. The interpretation of variants in nontranslated genes, such as *RMRP*, remains challenging because the standard variant classification criteria were developed for protein-coding genes and in vitro functional assays are limited. Evaluating *RMRP* variants requires integration of segregation data, population allele frequency, evolutionary conservation, and RNA structural analysis [11]. The variable clinical manifestations in CHH have been linked to the location of the variants and the degree of reduced rRNA or mRNA cleavage [12]. The 2 *RMRP* variants n.97_98dup and n.-25_-12dup identified in our patient have been previously reported in patients with CHH [4, 6, 13, 14], but this specific combination is novel. The n.-25_-12dup is located in the promoter region of *RMRP*, where other upstream insertions have been demonstrated to silence transcription and reduce gene expression [6]. The n.97_98 dup variant has shown to moderately decrease both rRNA and mRNA cleavage activity [12], which may explain the mild clinical presentation in our patient. However, previous studies have shown that the genotype does not always predict the severity of clinical symptoms and individuals with the same genotype may show different clinical features, even within families [15, 16].

Patients with CHH should be closely monitored for skeletal abnormalities, infections, anemia, and malignancies. Careful assessment of growth and development is essential because CHH may be associated with delayed or absent pubertal development [17]. Children and adolescents with CHH should undergo annual measurement of linear growth and body proportions using CHH-specific growth curves [9], with clinical assessment for musculoskeletal deformities at each visit and annual spine evaluation. Growth hormone treatment is generally not recommended [2]. Because the CHH growth failure results from a primary proliferation defect in growth plate chondrocytes, GH treatment is likely to result in no or only transient benefits, unless GH deficiency has been confirmed [18, 19]. The increased malignancy risk in CHH may need to be considered in treatment decisions [20]. Variable immune dysfunction may manifest at any stage, from infancy through late adolescence, or even adulthood [21]. Thorough clinical evaluation and assessment for recurrent infections is needed annually. Immunologic investigations at diagnosis should include total immunoglobulin and IgG subclass levels, white cell total and subclass level, and postvaccine titers. The most prevalent malignancies are non-Hodgkin lymphoma and basal cell carcinoma [22]; survival after lymphoma diagnosis is poor [23]. Thus, annual malignancy screening is important, including clinical evaluation, laboratory tests, and an abdominal ultrasound. Importantly, patients with pathogenic *RMRP* variants who have normal hair and lack extraskeletal manifestations in early childhood may still develop late-onset immunodeficiency and malignancies [24]. Therefore, continuous surveillance is crucial.

This study emphasizes the importance of recognizing skeletal dysplasia, including CHH, in children and adolescents with short stature to enable early diagnosis and proper management and to avoid unnecessary investigations. These patients require careful follow-up and early management of manifestations that may be associated with significant morbidity, including infections and malignancies.

## Learning points

- This case highlights the need for clinical awareness and consideration of skeletal dysplasia, such as CHH, in children with short stature to enable early diagnosis and proper management.
- Patients with CHH require careful follow-up and early management of manifestations that may be associated with significant morbidity, including infections and malignancies.
- Genetic screening for *RMRP* variants should be considered when CHH is suspected to enable accurate genetic counseling.

## Data Availability

Original data generated and analyzed during this study are included in this published article.
